# The Influence of Resident Level of Training on Fluoroscopy Time in Pediatric Supracondylar Humeral Fractures Treated with Closed Reduction and Percutaneous Pinning

**DOI:** 10.7759/cureus.2245

**Published:** 2018-02-28

**Authors:** Önder Kalenderer, Ali Turgut, Emre Bilgin, Serkan Erkus, Fikri B İpci, Tunc P Edizsoy

**Affiliations:** 1 Department of Orthopaedics and Traumatology, Tepecik Training and Research Hospital; 2 Department of Orthopaedics and Traumatology, Turan Turan Hospital

**Keywords:** supracondylar fracture, surgical experience, fluoroscopy, closed reduction, percutaneous pinning

## Abstract

Objective

The purpose of this study was to evaluate the effect of resident level of training on fluoroscopy duration in pediatric supracondylar humeral fractures treated with closed reduction and percutaneous pinning (CRPP).

Methods

After classifying the surgeons according to their seniorities, the duration of fluoroscopy time of 80 patients with extension type III supracondylar fractures during reduction and pinning was recorded.

Results

The time duration of reduction procedures was similar in all groups with respect to the surgical experience; however, time durations of percutaneous pinning procedures were found statistically different between groups (p=0.042).

Conclusion

Surgical experience is very important in the management of supracondylar humeral fractures in children, especially in percutaneous pinning rather than closed reduction.

## Introduction

Currently, the most common surgical procedure used for the treatment of pediatric supracondylar humeral fractures is closed reduction and percutaneous pinning (CRPP) [1]. Although CRPP has shown to be a safe, effective and reliable method, it is not without complications or disadvantages [2-3]. Low success rates were reported especially in type III extension fractures with very swollen elbows [4-5]. Furthermore, difficulty in full anatomic reduction and iatrogenic ulnar nerve injury has been reported with this treatment method [6]. Another disadvantage of this method is exposure to ionizing radiation both for the surgeon and the patient. Since fluoroscopic guidance has become a more prevalent practice in trauma surgeries today, more studies have recently been designed to investigate the exposure to radiation [7-11].

CRPP is one of the most common surgical procedures performed in orthopedic surgery. As a fundamental and basic procedure in general surgery, it is mainly performed by residents. CRPP procedure has a long learning curve [1]. Therefore, total fluoroscopy time, in other words radiation exposure, may change according to the resident's level of training in this procedure. A few previous studies have investigated the effect of surgical experience and fluoroscopy time in CRPP in pediatric supracondylar humeral fractures. However, these studies compared attending surgeons and residents. Currently, there is no study that compares the residents according to the level of training and duration of residency. The purpose of this study was to evaluate the influence of resident level of training on fluoroscopy duration in pediatric supracondylar humeral fractures treated with CRPP.

## Materials and methods

This retrospective study consisted of 80 patients with type III extension of supracondylar humeral fractures. None of these patients had arterial and/or nerve injuries. A long-arm cast was applied to all patients in the emergency room. After the hospitalization and preoperative preparation, patients underwent CRPP procedure under fluoroscopic guidance. The fluoroscopy equipment used at our institution, Tepecik Training and Research Hospital, is Philips BV Pulsera CE 0344 (Koninklijke Philips Electronics N.V., Holland). Fluoroscopy time and radiation dose emitted were available and printable in this equipment; these documents were added to the patient’s files and this data was used in this retrospective study.

All patients were operated under general anesthesia at a supine position on a radiolucent table. Anatomic reduction was confirmed by anterior-posterior and lateral fluoroscopic views when the elbow was at Jones position following classical reduction maneuvers. The duration of fluoroscopy time was recorded. Afterwards, first percutaneous pinning by 1.5-2.5 mm Kirschner wire was performed laterally. A second crossed Kirschner wire was inserted medially by reducing the flexion of the elbow. During the procedure, we used fluoroscopy guidance for proper insertions of the wires. The duration of fluoroscopy time during the pinning was also recorded and saved into the patients’ forms. After the surgery, a long arm cast was applied with the elbow flexed at 90º.

All surgical procedures in this series were performed by two residents training in orthopedics under the guidance of an attending orthopedic surgeon. The attending surgeons did not scrub and joined the operation. In our institute, in accordance with our residency curriculum, all residents training in orthopedics involve in surgical procedures of supracondylar humeral fractures as observers during their first two years of residency. After two years, they start to perform surgeries under the guidance of senior orthopedic surgeons. After three years, they perform surgery with two accompanying residents. Eight residents with different levels of training were included in the study. The most senior residents were a part of Group 1 while least senior residents were in Group 4. All residents were classified in these four groups by six-month periods according to their seniority. The least senior assistant was at three years of residency, while the most senior assistant was at 4.5 years of residency (Table 1).

**Table 1:** Number of residents in each group and their seniorities in orthopedics

	Number of Residents	Training time in Orthopedics
Group I	2	54 months
Group II	2	48 months
Group III	2	42 months
Group IV	2	36 months

We created a form and recorded the duration of fluoroscopy time that the surgeons were exposed to during the reduction and pinning procedures; the dosages of fluoroscopy were recorded in kilovolt and miliamper/seconds.

The data included patient demographics, the level of training of the surgeons in the procedure, fluoroscopy time(s), and radiation emission (millirems) as recorded from the fluoroscopy machine. Exclusion criteria included open fractures and neurovascular impairment.

Statistical analysis was performed with SPSS 21.0 software (IBM Corp., Armonk, NY, US). Mean duration of fluoroscopy of reduction and pinning of the surgeons were classified into four groups according to their seniorities. Kruskall Wallis test for four groups’ comparisons and Mann Whitney U test for two group’s comparisons were used. P values less than 0.05 were considered as statistically significant.

## Results

The mean age of the patients was seven (1-14) years. Fractures were reduced and percutaneously pinned at dosages of 46 kV (45–50, SD: 1.366) and 0.2 mA (Philips BV Pulsera CE 0344). Mean reduction time was 4.77±6.50 (1–29) seconds and mean percutaneous pinning duration time was 7.83±4.82 (1–24) seconds. Table 1 shows the duration of reduction to pinning stages according to the resident's level of training. The reduction durations were similar in four groups with respect to the surgical experience; however, percutaneous pinning durations were found to be statistically different between the groups (p=0.042) (Table 2).

**Table 2:** Duration of closed reduction and percutaneous pinning in four groups (seconds) *Kruskal Wallis Test

Groups	Group I (n=18) Mean±SD(min-max)	Group II (n=18) Mean±SD(min-max)	Group III (n=12) Mean±SD(min-max)	Group IV (n=32) Mean±SD(min-max)	Statistical Significance*
Closed Reduction	3.50±4.11(1–16)	3.39±4.55(1–20)	3.75±3.86(1–12)	6.63±8.71(1-29)	p> 0.05
Percutaneous Pinning	5.61±2.97(1–14)	6.39±3.45(3–14)	7.75 ±4.20(2–14)	9.91±5.75(1-24)	p = 0.042

The statistical significance of the two-group comparisons for the duration of percutaneous pinning is given in Table 3.

**Table 3:** The statistical significance of the two-group comparisons for the duration of percutaneous pinning Mann Whitney U Test

	Group I	Group II	Group III	Group IV
Group I		p> 0.05	p> 0.05	p =0.012
Group II	p> 0.05		p> 0.05	p =0.047
Group III	p> 0.05	p> 0.05		p> 0.05
Group IV	p =0.012	p =0.047	p> 0.05	

## Discussion

In orthopedic surgery, the usage of fluoroscopy has been gradually increasing as minimally invasive surgical procedures come forward and the exposure of both patients and surgical staff to ionizing radiation increases with such procedures [7-9]. Fluoroscopy is frequently used in the treatment of supracondylar humeral fractures in children. Although there are various surgical treatment methods, CRPP has been widely advocated and preferred method due to its excellent results. In this study, we investigated the fluoroscopy time during the surgery of type III supracondylar (extension type) fractures. The exposure time and grade of ionizing radiation were affected by the preferred treatment option, safety protection, intermittent utilization of fluoroscopy, difficulty level of the fracture, the experience of the surgical staff, and the distance between the target and the radiation source [7-8, 10-11].

In pediatric supracondylar humeral fractures, the treatment strategy is the most important factor that affects the fluoroscopy time and exposed radiation dose. In a recent study, comparing the open and closed reduction procedures, three times more exposure to radiation was determined in closed reduction procedure [12]. In another study, Kraus et al. reported that the average intraoperative image intensifier time was 30.7 s (9-121) for K-wire procedure, 41.4 s (13-94) for external fixator, and 80.0 s (18-228) for ESIN (11). In another study, external fixation and intramedullary pinning were compared and exposure time to radiation was reported at 84 seconds in the external fixation group and 156 seconds in the intramedullary pinning group [13]. These reported time periods revealed that there is more exposure to radiation both in fixation and intramedullary pinning procedures than open and closed reduction and internal fixation. In our study, in 80 patients and four different groups, mean fluoroscopy exposure time was measured as 13.45 seconds (2-48).

The level of training of the surgeon was also a major factor in the reduction of radiation with the presence of a senior surgeon causing a 40% decrease in amount of radiation [13]. This finding is similar to that of Blattert et al. who found a 36% decrease in radiation exposure time in procedures performed by the senior staff. As surgical experience increases, exposure time to radiation was found to decrease [14]. Liu et al. recommended a backup surgeon for the first 15 cases of supracondylar humeral fractures treated by pediatric orthopedic fellows [15].

In our study, both CRPP time periods were also evaluated separately. The time of closed reduction maneuver was found similar between all residents; however, internal fixation procedure was found different. As the resident's level of training increases, it takes less time for fixation of the fracture by K-wire. The most important factor that increased the total fluoroscopic exposure time was the pining procedure in supracondylar fractures.

## Conclusions

This is the first study that evaluates the radiation exposure time in CRPP of pediatric humeral supracondylar fractures performed by residents. The results of our study showed that percutaneous pinning procedure has a longer learning curve than closed reduction procedure. Therefore, orthopedic training programs for residents may be arranged to develop this skill.
